# Dynamics of Tilapia Lake Virus in Recirculating Aquaculture Systems and the Impact of Vaccination on Outbreak Control

**DOI:** 10.3390/v18010096

**Published:** 2026-01-09

**Authors:** Montakarn Sresung, Jidapa Yamkasem, Supitchaya Theplhar, Piyathip Setthawong, Surapong Rattanakul, Skorn Mongkolsuk, Kwanrawee Sirikanchana, Win Surachetpong, Tuchakorn Lertwanakarn

**Affiliations:** 1 Graduate Program in Animal Health and Biomedical Science, Faculty of Veterinary Medicine, Kasetsart University, Bangkok 10900, Thailand; montakarn.sr@ku.th; 2 Department of Physiology, Faculty of Veterinary Medicine, Kasetsart University, Bangkok 10900, Thailand; jidapa.yam@ku.th (J.Y.); supitchaya.theplhar@gmail.com (S.T.); piyathip.s@ku.th (P.S.); 3 Research Laboratory of Biotechnology, Chulabhorn Research Institute, Bangkok 10210, Thailand; skorn@cri.or.th (S.M.); kwanrawee@cri.or.th (K.S.); 4 Department of Environmental Engineering, Faculty of Engineering, King Mongkut’s University of Technology Thonburi, Bangkok 10140, Thailand; surapong.rat@kmutt.ac.th; 5 Institute of Aquaculture, Faculty of Natural Sciences, University of Stirling, Stirling FK9 4LA, UK

**Keywords:** water monitoring, recirculating aquaculture system, droplet digital PCR, vaccine, tilapia

## Abstract

Tilapia lake virus (TiLV) is a highly virulent pathogen that has caused substantial mortality in tilapia farms, particularly those with open-water systems. However, TiLV can also emerge and persist in closed environments, such as recirculating aquaculture systems (RAS), where environmental accumulation and repeated exposure may intensify infection and sustain outbreaks. In this case study, we conducted three field experiments to better understand TiLV dynamics among Nile tilapia in RAS. In experiment I, we quantified the TiLV levels in the fish, water, and sediment to compare outbreak and no-outbreak conditions and found that the TiLV concentrations in liver samples and the water were significantly higher in the outbreak ponds and positively correlated with increased fish mortality. In experiment II, we used a side-by-side field trial to evaluate the protective efficacy of a TiLV vaccine and its effects on the viral loads in the fish and aquatic environment during outbreaks. The vaccinated fish showed substantially lower cumulative mortality (16.7%) than the unvaccinated controls (37.7%), with a relative percent survival of 55.6%. Additionally, the TiLV concentrations in the pond water of the vaccinated group were significantly lower. In experiment III, we compared the TiLV patterns between RAS and non-RAS operations to determine how water recirculation influences viral accumulation and outbreak severity. The results revealed limited viral accumulation and shorter disease outbreak duration in the non-RAS. Overall, our findings showed that the TiLV levels in the rearing water were closely linked with disease severity in the RAS-based tilapia hatcheries. Continuous water recirculation allowed the virus to build up in the system, which led to more prolonged outbreaks, while the non-RAS conditions with regular water discharge showed lower viral loads and faster recovery. The vaccinated fish had better survival rates and released less virus into the water, which helped reduce infection pressure across the ponds. Together, these results suggest that combining vaccination with good water management and molecular monitoring can provide a practical, noninvasive way to detect and control TiLV outbreaks in intensive farming systems.

## 1. Introduction

Tilapia lake virus (TiLV), officially designated as *Tilapinevirus tilapiae*, has emerged as a major threat to global tilapia aquaculture [1]. The virus is associated with mass mortality among fish as well as substantial economic losses, particularly in low- and middle-income countries where tilapia is a primary source of animal protein [2,3]. Mortality in TiLV-affected populations is highly variable—from 20% to more than 90%—which reflects differences in host susceptibility, environmental conditions, coinfecting pathogens, and farm management practices [4,5,6,7,8]. Since its first detection in Israel in 2014, TiLV has been reported in more than 18 countries [9,10], which has raised concerns about its transboundary spread and the vulnerability of both hatchery and grow-out systems [5,11,12]. TiLV transmission occurs both vertically from infected broodstock to offspring [13] and horizontally through direct contact or cohabitation with infected fish [8,14]. Although evidence regarding water-mediated infection is limited, TiLV RNA and infectious particles have been detected in water, fish mucus, and fecal materials, which suggests that the aquatic environment may serve as a reservoir for viral particles occurrence and the accumulation of pathogens [15,16]. The results of experimental studies have further suggested that TiLV can remain viable in water for at least 24 h, while viral RNA may persist for up to seven days post-excretion, thereby highlighting the potential of environment persistence of the virus and fish re-exposure to TiLV under intensive conditions [17,18].

In intensive aquaculture systems, such as recirculating aquaculture systems (RAS), viral particles shed via mucus, feces, or decomposing tissues can accumulate in water and sediment and, if viable, increase the overall infection pressure [19,20]. These environmental reservoirs may therefore sustain infection within ponds, particularly under conditions of limited water exchange and inadequate disinfection. The rationale for the present case study arose from an investigation at a commercial tilapia nursery farm in Kalasin province, Thailand, where persistently high mortality rates had been experienced across multiple production cycles. The outbreaks were closely associated with the monthly introduction of new fish stocks, for which adequate quarantines were not being conducted. The farm operated a closed-loop RAS equipped with ultraviolet (UV) disinfection, a commonly used method to improve water quality in aquaculture facilities [21]. However, the use of UV irradiation may only inactivate pathogens rather than eliminate them or substantially reduce their loads within the system [22,23]. Despite these measures, disease outbreaks recurred and were subsequently confirmed to have been caused by TiLV.

Vaccination is considered one of the most effective strategies for controlling infectious diseases in fish, as it stimulates adaptive immune responses, reduces systemic viral replication, and improves survival. Beyond reducing mortality, vaccination can lower viral shedding from infected fish and thereby decrease the risk of infection pressure and limit viral buildup in the aquatic environment [24,25]. Several vaccine platforms, including inactivated, live attenuated, and recombinant formulations, have been developed for TiLV, and these have achieved relative percent survival (RPS) values of 50–85% depending on the delivery route and antigen design [26,27]. Nevertheless, field-based evaluations of TiLV vaccine efficacy under commercial farming conditions remain scarce. Studies have shown that both whole inactivated virus and recombinant segment 4, encoding the nucleoprotein of the virus, vaccines administered via immersion have improved fish survival in net-cage culture systems [27,28]. However, the extent to which vaccination influences environmental viral loads under real-world RAS conditions is not well understood. In this study, we therefore aimed:

(1) to determine whether TiLV levels in rearing water can serve as a reliable, noninvasive indicator of infection intensity and outbreak progression,
(2) to evaluate the protective efficacy of a TiLV vaccine under outbreak conditions in a RAS-operated tilapia farm,
(3) to compare TiLV occurrence and outbreak patterns between RAS and non-RAS operations to clarify the role of water recirculation in viral accumulation and infection pressure.

## 2. Materials and Methods

### 2.1. Study Design and Sample Collection

This study was conducted at a commercial tilapia nursery farm located in Kalasin province, Thailand. The facility comprises 24 indoor concrete ponds (size 100 m^3^) housed with two enclosed nursery buildings operating under RAS, with a separate water treatment building for filtration and disinfection. The farm introduces a new batch of Nile tilapia fry monthly. Each batch consists of approximately 53,000 fries, which are sourced from an external hatchery and have an initial weight of 0.40 g, a stocking density of 0.11 kg/m^3^. The fish are reared for approximately 50–60 days and fed twice at 3% of the body weight until they reach a weight of 10 g, at which time they are moved to the grow-out cages. The RAS is equipped with a UV disinfection unit using 254 nm wavelength lamps with an average applied UV dose of 50 mJ/cm^2^, which is within the range commonly recommended for effective microbial inactivation in water treatment, as doses above ~25–30 mJ/cm^2^ are typically used to achieve significant pathogen inactivation in similar systems [29,30]. The farm’s water treatment system comprises multiple stages of water quality improvement, including biological filtration for the removal of ammonia and nitrite, aeration to increase dissolved oxygen concentrations and enhance nitrification efficiency for the conversion of nitrite to nitrate, sedimentation to reduce suspended solids and organic matter, and the addition of make-up water to maintain overall water quality stability, prior to the UV disinfection unit. The water quality parameters including daily temperature, dissolved oxygen, pH, and ammonia-nitrogen levels were monitored and remained within normal limits throughout the study. The water exchange rate was set as 51.59 m^3^/h, and the photoperiod was set as 12 h light/12 h ht/dark. We conducted three experiments to assess the infection dynamics, vaccine efficacy, and environmental viral loads during different outbreak scenarios.

#### 2.1.1. Experiment I—Comparison of Outbreak and No-Outbreak Batches

The first experiment was designed to compare the TiLV loads in the fish, water, and sediment in a production batch without an outbreak and one with a confirmed TiLV-associated outbreak. Control ponds (no outbreak) and outbreak ponds were located in the same building but were spatially distant and managed with separate equipment to minimize cross contamination risk. Sequential samples were collected repeatedly from the same selected ponds throughout the experiment to ensure consistency in temporal comparisons. Samples of the fish, water, and sediment were collected from the two production batches as follows:

(i) Outbreak condition: During a suspected TiLV outbreak, sampling of the affected production batch was initiated once the daily mortality rate exceeded 1% of the total fish. Event-based sampling was subsequently conducted on days 1, 4, 7, and 14 following this mortality threshold, which corresponded to days 26, 29, 32, and 39 post-stocking. The samples were randomly collected from four of the 10 production ponds. At each sampling event, water and sediment samples and a pooled liver from five moribund or freshly dead fish were collected once from each pond, totaling 16 water, 16 sediments, and 16 pooled fish samples over the course of the study. All the samples were stored at 4 °C until RNA extraction for further TiLV genomic quantification. The TiLV infection was confirmed by the relevant clinical signs such as skin erosion, abdominal distension, and exophthalmia and RT-qPCR results. Notably, routine health monitoring at the farm did not detect significant outbreaks of common bacteria (e.g., *Streptococcus* spp., *Aeromonas* spp.) or parasitic pathogens that could explain the observed mortality scale. One water sample was also collected from each filtration system outlet to determine the TiLV concentration on the day prior to fry stocking. These samples were used as the baselines to compare the initial TiLV loads with those in the ponds during the outbreak period.
(ii) No-outbreak condition: Sampling of the no-outbreak batches was conducted on days 37, 39, and 46 post-stocking, at which times the fish exhibited no clinical signs or daily mortality. Four of 10 rearing ponds were randomly sampled, and one water and one sediment sample along with a pooled liver sample from five fish were randomly collected from each pond at each time point, with a total of 12 fish, 12 sediments and 12 pooled liver samples per group throughout the study. One additional baseline water sample was collected from each filtration system outlet on the day before the introduction of each new batch of fry.

#### 2.1.2. Experiment II—Evaluation of the Vaccine Efficacy During TiLV Outbreak in RAS

The objective of this experiment was to evaluate the field efficacy of a previously described chitosan nanoparticle-based immersion recombinant protein vaccine targeting TiLV segment 4 (CNS4) [27,28] under commercial RAS conditions. For this assessment, we focused on the cumulative mortality of the fish and TiLV viral loads in the fish and environmental (water and sediment) samples. Two experimental groups of fish were reared side by side under identical farming conditions: (i) unvaccinated (*n* = 26,500) and (ii) vaccinated fish (*n* = 26,500). Each group was evenly distributed across five ponds (5300 fish per pond). All selected ponds had a similar history of production and were subjected to standard cleaning, disinfection, and drying procedures before the start of the new crop. Treatment (vaccinated) and control (unvaccinated) ponds were allocated using a randomized block design within the same building to ensure they experienced similar environmental conditions while minimizing spatial clustering effects and potential confounders. The sample collection was initiated upon detection of increased mortality exceeding 1% in either group. The samples were thus collected on days 1, 4, 7, and 14 following the onset of the outbreaks, which corresponded to days 33, 36, 39, and 46 post-stocking. At each sampling event, two of the five ponds were randomly selected per group (unvaccinated and vaccinated fish). One water sample, one sediment sample, and a pooled liver from five fish were collected from each selected pond at each time point. Over the course of the study, this resulted in 8 water, 8 sediment, and 8 pooled fish samples per group. In addition, one baseline water sample was collected at the outlet of each filtration system on the day prior to fry stocking. The vaccine efficacy was expressed as RPS, which was calculated using the following formula:

(1) $$RPS=(1-\frac{Cumulative\;mortality\;in\;vaccinated\;group}{Cumulative\;mortality\;in\;unvaccinated\;group})\times 100\%$$

#### 2.1.3. Experiment III—Investigation of TiLV in the Environment During Non-RAS Operation

In this experiment, we aimed to evaluate the TiLV viral loads during a natural outbreak in a non-RAS condition. In contrast to experiments I and II, the water from the rearing ponds was discharged without being recirculated. Two of the 10 production ponds were randomly selected and consistently sampled across all the time points. Fish, water, and sediment samples were collected on days 1, 4, 7, and 14 following the initial observation of mortality exceeding 1%, which corresponded to days 12, 15, 18, and 25 post-stocking. At each time point, two water and two sediment samples and ten individual fish were collected from each pond, resulting in 80 fish samples for this condition. In addition, one baseline water sample was collected from the filtration system outletprior to fry introduction.

### 2.2. RNA Extraction and Complementary DNA Synthesis

Water samples (1 L) were collected in sterilized polypropylene bottles from the effluent discharge pipe of each rearing pond just prior to wastewater entry. Sediment samples (1–2 g) were collected using sterile plastic spoons from the pond sediment filters and transferred into sterile 50 mL polypropylene tubes. The water samples were processed using an adsorption-extraction method modified from [18]. In brief, a 500 mL aliquot of each water sample was filtered through a 0.45 µm pore-size mixed cellulose esters HAWP membrane (Merck, Darmstadt, Germany) using a vacuum filtration system. The membrane was then cut into small pieces for RNA extraction. To ensure procedure integrity and monitor potential cross-contamination, method blanks were included by filtering 500 mL of sterile reverse osmosis water following the same procedure [31]. Both the filter membrane and sediment samples (200 mg) were extracted using the Quick-RNA^TM^ Fecal/Soil Microbe Microprep Kit (Zymo Research, Irvine, CA, USA) according to the manufacturer’s instructions. The samples were homogenized in ZR BashingBead^TM^ lysis tubes with an S/F RNA lysis buffer for 10 min. The total RNA was eluted in 30 µL of elution buffer, and its concentration and purity were assessed using a NanoDrop^TM^ 2000 spectrophotometer (Thermo Scientific, Waltham, MA, USA). All the extracted RNA was stored at −20 °C and processed for cDNA synthesis within 48 h.

For the fish samples, pooled liver tissue (100 mg) from five fish was subjected to RNA extraction using the phenol-chloroform method with GENEzol^TM^ Reagent (Geneaid, New Taipei, Taiwan). Tissue homogenization was performed in 1 mL of GENEzol^TM^, followed by the addition of 200 µL chloroform. After vortexing, the mixture was centrifuged at 4 °C for 15 min, and the aqueous phase was collected and mixed with an equal volume of isopropanol, then incubated at −20 °C for 4 h to facilitate RNA precipitation. The RNA pellet was recovered following centrifugation, washed with 70% ethanol, air-dried, and resuspended in 50 µL of diethyl pyrocarbonate-treated water. The samples were stored at −20 °C until use. The cDNA was synthesized using the ReverTra Ace^®^ cDNA synthesis kit (Toyobo, Osaka, Japan) in line with the manufacturer’s protocol. Each 20 µL reaction contained 4 µL of 5× reverse transcriptase (RT) buffer, 1 µL of primer mix (1250 nM random primer, 500 nM Oligo (dT), and 500 nM specific primer), 1 µL of RT mix, 2 µL RNA template, and nuclease-free water. The reverse transcription was performed at 42 °C for 60 min, followed by an enzyme inactivation step at 98 °C for 5 min using a Mastercycler^®^ nexus Thermal Cycler (Eppendorf, Hamburg, Germany). The synthesized cDNA was stored at 4 °C until droplet digital PCR (ddPCR) analysis.

### 2.3. ddPCR for TiLV Detection

We used the QX200^TM^ ddPCR system (Bio-Rad, Hercules, CA, USA) to detect and quantify the TiLV. Each reaction mixture contained 10 μL of 2× EvaGreen^®^ Supermix (Bio-Rad, Hercules, CA, USA), 0.2 μL each of 10 μM forward and reverse primers TiLV_112F (5′-CTGAGCTAAAGAGGCAATATGGATT-3′) and TiLV_112R (5′-CGTGCGTACTCGTTCAGTATAAGTTCT-3′) [16], 2 μL cDNA template, and nuclease-free water to reach a final volume of 20 μL. The reaction mix was briefly vortexed, then partitioned into ~10,000 nanoliter-sized droplets using a QX200^TM^ Droplet Generator (Bio-Rad, Hercules, CA, USA). For droplet generation, 20 μL of the prepared reaction mixture was loaded into the sample wells of DG8^TM^ cartridges (Bio-Rad, Hercules, CA, USA) followed by the addition 70 μL of QX200^TM^ droplet generation oil for EvaGreen into the oil wells of the same cartridges. Droplets were generated and transferred to a 96-well plate. The plate was sealed using a PX1^TM^ PCR Plate Sealer (Bio-Rad, Hercules, CA, USA). PCR amplification was conducted using a T100^TM^ Thermal Cycler (Bio-Rad, Hercules, CA, USA) under the following cycling conditions: initial enzyme activation at 95 °C for 5 min, 40 cycles of denaturation at 95 °C for 30 s, annealing at 56 °C for 3 s, and a final extension at 98 °C for 10 min. A ramp rate of 2 °C/s was applied per second throughout the process to maintain the droplet integrity. Post-amplification, the droplet fluorescence was read using the QX200^TM^ droplet reader (Bio-Rad, Hercules, CA, USA), and the data were analyzed using QuantaSoft^TM^ software (Bio-Rad, Hercules, CA, USA). The TiLV copy numbers were calculated using Poisson statistics and expressed as copies per µL. As recommended by the manufacturer, only the reactions with more than 10,000 droplets were included in the final analysis to ensure data accuracy and statistical reliability [32].

### 2.4. Statistical Analysis

All the statistical analyses were conducted using R software version 4.3.3 [33]. The datasets included the viral loads measured in the fish liver tissue, water, and sediment samples as well as the cumulative mortality rates of the fish from each group. For the datasets in which all the values were above the limit of detection (LOD) (i.e., fully positive datasets), the data normality was assessed using the Shapiro–Wilk test. If the data followed a Gaussian distribution, independent two-group comparisons were undertaken using two-sample *t*-tests. For the non-normally distributed data, the Wilcoxon rank-sum test was applied for independent two-group comparisons. We compared the TiLV levels in the ponds on different days for each group using repeated measures ANOVA followed by Tukey’s multiple comparisons test.

For the datasets that contained values below the LOD, referred to as non-detects, no imputation or substitutions (e.g., zeros or LOD ÷ 2) were applied to avoid bias and preserve the integrity of the data [34]. Instead, a nonparametric survival analysis approach was employed using the Kaplan–Meier estimator with Efron’s bias correction [34,35], which is specifically designed to handle left-censored data (non-detects) without data loss or distortion. Normality was assessed using the Shapiro–Wilk test for datasets with a single LOD value, while the Shapiro–Francia test was applied for datasets with multiple LOD levels.

Two group comparisons involving censored data were conducted using the generalized Wilcoxon test adjusted with Holm’s correction. To ensure the accuracy of the correlation analysis when handling the censored datasets, all the data (including the non-detects) were ranked using the U-score ranking method. This approach enables reliable analysis even when numerical values are identical or fall below the LOD and thus preserves the rank order distinction between detected and non-detected observations. Finally, Spearman’s rank correlation analysis was performed to assess the relationships between the TiLV viral loads in the water, sediment, and fish liver tissue and the cumulative mortality of the fish under the RAS conditions in experiments I and II.

## 3. Results

### 3.1. Mortality Patterns and TiLV RNA Concentrations Under Outbreak and No-Outbreak Conditions in a RAS Setup

**Figure 1:**
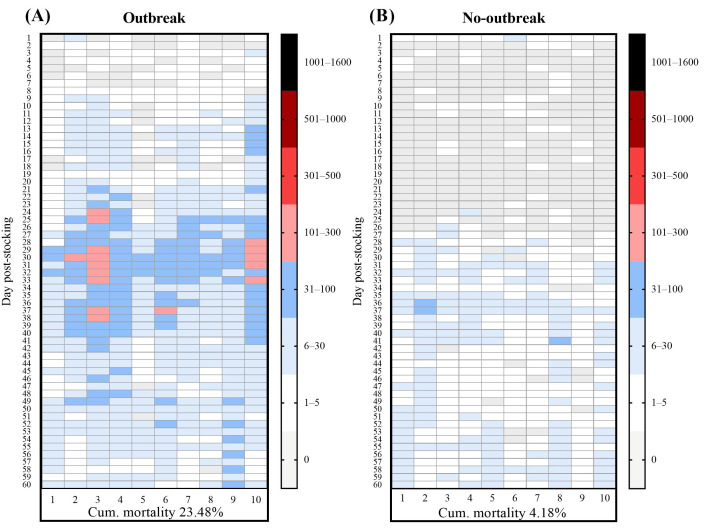
Mortality patterns of Nile tilapia in (**A**) tilapia lake virus (TiLV) outbreak and (**B**) no-outbreak batches over a 60-day nursery period. The daily mortality data were collected from 10 nursery ponds (*n* = 5300 fish per pond). Each tile in the heatmap represents the number of fish mortalities per day in a single pond. The color scale indicates the daily mortality intensity per pond.

Given the recurring mortality and TiLV outbreaks previously observed at the tilapia RAS farm, we first characterize the outbreak pattern and quantify the TiLV RNA concentration in fish and environmental samples. To achieve this, we compared production cycles that showed fish with TiLV-like clinical signs with those where the fish remained healthy. Our results revealed clear differences in the clinical presentation and mortality patterns between the TiLV-outbreak and no-outbreak production batches reared using an identical RAS. In the outbreak batches, the infected fish showed typical TiLV-associated signs, including abnormal swimming, lethargy, skin pallor, and fin erosion. Daily mortality began exceeding 1% of the total stock around 25 days post-stocking (dps) and peaked near 30 dps, with a mean cumulative mortality of 23.48% ± 3.60% (Figure 1A). In contrast, the fish from the no-outbreak batches remained clinically normal throughout the production cycle. No signs of disease were observed, and daily mortality remained stable with a cumulative mortality of 4.18% ± 0.54% (Figure 1B). Notably, detection of TiLV RNA from the pooled liver samples of both outbreak and no-outbreak batches was 87.5% (14/16) and 58.3% (7/12), respectively (Table 1). However, the viral RNA concentrations in the no-outbreak batch were substantially lower (*p* = 0.0438) than the outbreak group (Table 1).

The TiLV RNA concentrations in the water and sediment from the rearing ponds of the outbreak and no-outbreak batches were compared (Table 1). In the outbreak batches, TiLV RNA was detected in all the water samples (100%, 16/16) (Table 1). TiLV was similarly detected in most of the water samples from the no-outbreak batches (91.7%, 11/12) but at significantly lower concentrations (*p* = 0.0223). The viral RNA concentrations in the sediment samples of the outbreak batches remained relatively stable. When we compared the viral RNA concentrations in the sediment between the outbreak and no-outbreak batches, the difference was not statistically significant.

### 3.2. Mortality Patterns and TiLV RNA Concentrations Under Vaccinated and Unvaccinated Conditions

**Figure 2:**
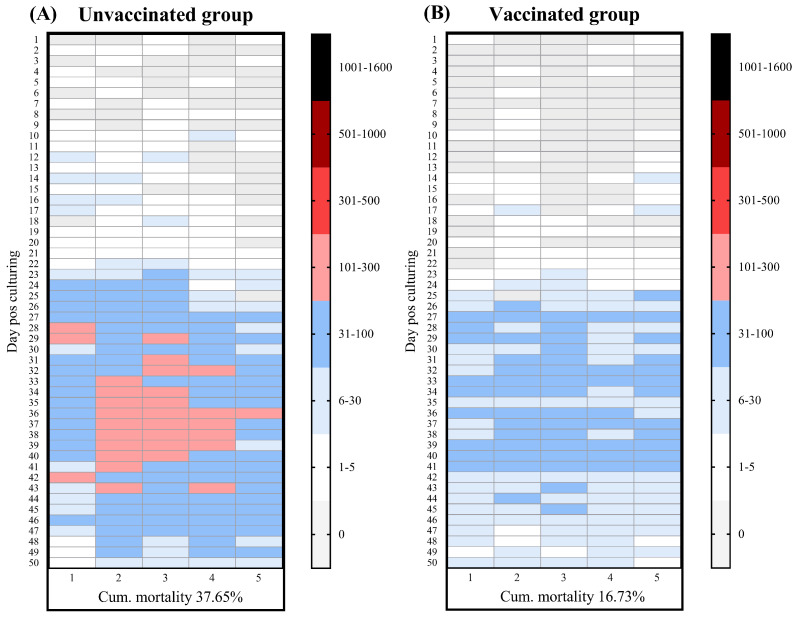
Mortality patterns of (**A**) unvaccinated and (**B**) vaccinated groups during a TiLV outbreak over a 50-day nursery period. The fish were reared side by side under identical recirculating aquaculture system (RAS) conditions. Each tile in the heatmap represents the number of fish mortalities per day within an individual pond, and the color scale indicates the relative daily mortality intensity per pond.

Building upon the findings in Section 3.1, we evaluated whether vaccination could mitigate disease and lower environmental TiLV RNA concentrations. Specifically, the efficacy of the CNS4 vaccine [27] was assessed by comparing the disease severity, mortality patterns, and viral RNA concentrations between the vaccinated and unvaccinated groups during TiLV outbreaks (Figure 2A,B). In the unvaccinated group, clinical signs and mortality became apparent at 24 dps and persisted until the end of the production cycle, with a cumulative mortality of 37.65% ± 4.91% (Figure 2A; Table 2). In contrast, the vaccinated group showed a delayed infection onset and shorter duration of mortality (between 27 and 41 dps), with a significantly lower cumulative mortality of 16.73% ± 1.03% (*p* = 0.0090) (Figure 2B). The calculated RPS in the vaccine group was 55.57%. Molecular analysis confirmed the presence of TiLV in the pooled liver samples from both groups, with detection rates of 100% in the unvaccinated group and 87.5% in the vaccinated group. Notably, the mean viral RNA concentrations in the vaccinated group were lower than that in the unvaccinated group, although this difference was not statistically significant (*p* = 0.1172) (Table 2).

To further evaluate the impact of vaccination on environmental shedding, the TiLV RNA concentrations in the water and sediment between the vaccinated and unvaccinated groups were compared (Table 2). In the unvaccinated group, TiLV RNA was detected in all the water samples (100%, 8/8). Meanwhile, TiLV RNA was detected in 87.5% (7/8) of the water samples from the vaccinated group but at a significantly lower mean concentration compared with the unvaccinated group (*p* = 0.0039). In the sediment samples, however, no significant differences were observed between the two groups.

### 3.3. Mortality Patterns and TiLV RNA Concentrations During TiLV Outbreaks in a Non-RAS Condition

**Figure 3:**
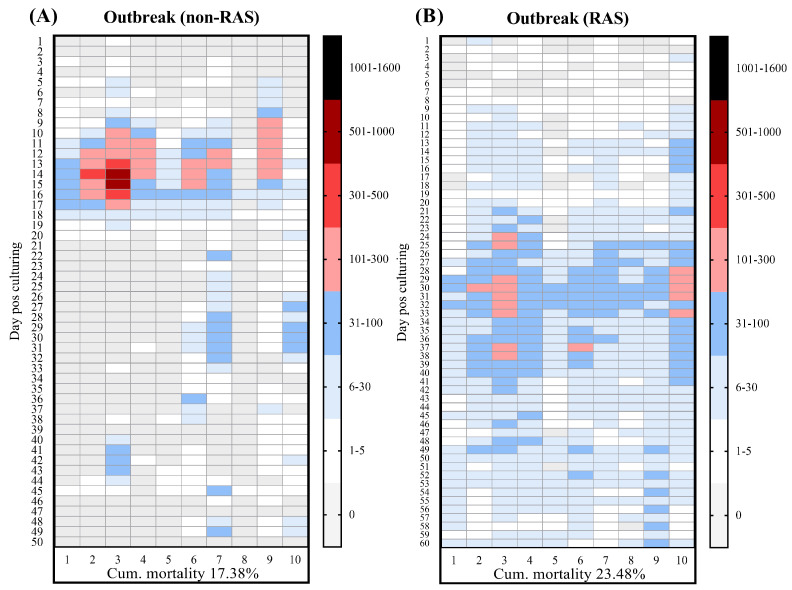
Mortality patterns of Nile tilapia during a TiLV outbreak under (**A**) non-RAS and (**B**) RAS conditions over the production cycle. The daily mortality in 10 nursery ponds (*n* = 5300 fish per pond) was recorded. Each tile in the heatmap represents the number of fish mortalities per day within an individual pond, and the color scale indicates the relative intensity of daily mortality per pond.

To examine the influence of water recirculation on TiLV dynamics, we applied a non-RAS approach by discontinuing water reuse and allowing the continuous discharge of water from the rearing ponds. The mortality pattern in the non-RAS condition contrasted with that observed under the RAS condition (Figure 3). The onset of the outbreak in the non-RAS occurred earlier (between 11 and 17 dps), with a cumulative mortality of 17.38% ± 4.74%. This trend was supported by molecular analysis, which detected TiLV RNA in 93.8% (75/80) of the liver samples from individual fish in the non-RAS (Table 3). Although the overall mortality in the non-RAS condition was numerically lower than that in the RAS setup, the difference was not statistically significant (*p* = 0.3442). Environmental testing showed lower TiLV detection frequencies for the non-RAS condition compared with the RAS condition. Specifically, TiLV RNA was detected in 50% (4/8) of the non-RAS water samples and 87.5% (7/8) of the non-RAS sediment samples (Table 3). Notably, the TiLV concentrations in the water were significantly reduced in the non-RAS condition compared with those in the RAS condition (*p* = 0.0064), whereas the TiLV concentrations in the sediment were statistically similar between the two conditions.

### 3.4. Associations Between TiLV RNA Concentrations in Water and Other Parameters in the Three Experimental Conditions

**Figure 4:**
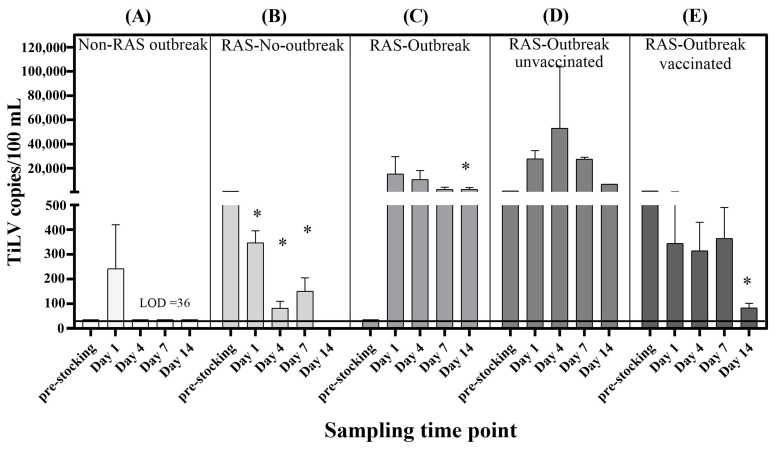
TiLV concentrations in the baseline (pre-stocking) and rearing pond water in (**A**) non-recirculating aquaculture system (RAS) with outbreak, (**B**) RAS with no-outbreak, (**C**) RAS with outbreak, (**D**) RAS with outbreak and unvaccinated and (**E**) RAS with outbreak and vaccinated batches. For the no-outbreak group (**B**), samples were collected in line with the farm’s historical baseline schedule. The bars represent the mean values, and the error bars indicate the SEM. The dashed line represents the LOD: 36 copies per 100 mL. Asterisks (*) indicate significant differences (*p* < 0.05) compared with the pre-stocking values. collected on days 1, 4, 7, and 14 after the onset of outbreak.

The TiLV concentrations in the pond water from all the production batches were compared throughout the rearing period relative to the respective pre-stocking baseline levels (Figure 4, Supplemenatary Table S1). At baseline, the TiLV concentrations varied modestly from 36–1350 copies per 100 mL. In the non-RAS batch (Figure 4A), the pre-stocking water contained viral RNA concentrations below the LOD, and the TiLV concentrations remained consistently low throughout the production cycle. In contrast, the pre-stocking water from the RAS batches contained detectable TiLV RNA at levels higher than those observed in the non-RAS batches (Figure 4B–D). In two independent RAS outbreak batches (Figure 4C,D), in which both the cumulative mortality of the fish and the TiLV loads in the pooled liver samples were significantly elevated, the TiLV concentrations in the water increased sharply from days 1 to 14 post-outbreak and reached 2702–53,150 copies per 100 mL. These concentrations were substantially higher than those measured in the corresponding pre-stocking baseline samples (36–1350 copies per 100 mL) and the no-outbreak RAS batches (83–346.7 copies per 100 mL) (Figure 4B). The water samples collected from the vaccinated RAS batch (Figure 4E) exhibited consistently lower TiLV concentrations compared with those from the RAS ponds with unvaccinated groups (Figure 4D) under identical outbreak conditions. Notably, the concentrations of TiLV in the water mirrored the trends observed for the liver tissue samples and the cumulative mortality of the fish in both the vaccinated and unvaccinated groups.

**Figure 5:**
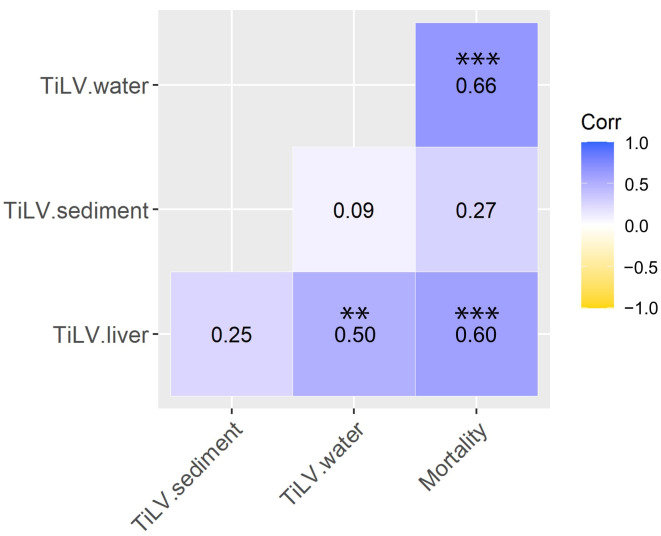
Spearman’s rank correlation analysis of the TiLV concentrations in the water, sediment, and liver tissue samples and fish mortality under the RAS condition. The values represent Spearman’s rho (*ρ*) correlation coefficients, with the color scale indicating the correlation level. Asterisks indicate statistically significant correlations, where ** denotes *p* < 0.01 and *** signifies *p* < 0.001.

Spearman correlation analyses further revealed significant positive relationships between the TiLV RNA concentrations in the water, sediment, liver tissue, and fish mortality under the RAS condition (Figure 5). Specifically, the viral RNA concentrations in the water correlated with fish mortality (*ρ* = 0.66; *p* < 0.001) and viral loads in the liver tissue (*ρ* = 0.50; *p* < 0.01). A similar correlation was found between the viral RNA concentrations in the liver tissue and fish mortality (*ρ* = 0.60; *p* < 0.001). In contrast, the TiLV RNA concentrations in the sediment did not show any significant or consistent relationship with either the liver tissue viral loads or fish mortality.

## 4. Discussion

RAS have been increasingly adopted in commercial tilapia farming to minimize water consumption, reduce effluent discharge [36,37,38], and enhance biosecurity practices by limiting the introduction and spread of pathogens [39,40]. However, inadequate disinfection or suboptimal water treatment may allow infectious agents to accumulate and recirculate within the RAS production loop. In this study, we investigated a TiLV-endemic commercial tilapia nursery farm that uses a RAS and demonstrated that recirculating water can function as a viral reservoir for TiLV RNA that promotes viral accumulation and repeated exposure across production cycles. Indeed, TiLV RNA was consistently detected in the pre-stocking water samples, which indicated incomplete viral clearance prior to fry introduction, and these viral concentrations increased rapidly during the onset of outbreaks, which coincided with elevated fish mortality and clinical signs. Additionally, we observed clear correlations between the TiLV concentrations in the rearing water, outbreak severity, and viral loads in the liver tissue samples from all experiment. These results suggested that the RAS conditions may facilitate the retention of TiLV RNA in water which may be associated with disease progression and fish mortality.

### 4.1. Dynamics of Environmental TiLV During the Outbreak in RAS-Operated Farm

During the outbreak periods in our study, when the daily mortality increased sharply, the TiLV concentrations in the rearing water rose concurrently, which was consistent with active viral shedding and increasing infection pressure within the system. In contrast, the TiLV concentrations in the sediment showed no significant correlation with either the fish mortality or the viral loads in the liver samples and not differ significantly between outbreak and nooutbreak periods. The relatively low TiLV levels in the sediment could be partly explained by multiple factors affecting the binding affinity of TiLV of viral particles including the enveloped nature of TiLV. Similar observations have been reported for other enveloped viruses such as SARS-CoV-2 [41] and respiratory syncytial virus [42]. Moreover, the physiochemical interactions between viral particles and suspended solids, particularly the surface charge of viruses and adsorption properties, can influence virus–particle interactions, which may affect viral persistence and detectability during the environmental sampling [43]. Despite these differences, environmental detection of pathogen genomic DNA or RNA in water has become increasingly recognized as a valuable tool for disease surveillance in aquaculture [44]. For example, recent studies have demonstrated the application of such water-based monitoring approaches for tracking infectious spleen and kidney necrosis virus [45], *Flavobacterium psychrophilum*, *Yersinia ruckeri* [46], *Aeromonas veronii* [20], *P. damselae piscicida*, *Vibrio harveyi*, and *Vibrio anguillarum* [47]. Together with those of previous publications, our findings highlight the potential of environmental TiLV detection as a reliable, noninvasive approach for establishing early warning systems in intensive farming practices, tracking infection dynamics, and supporting farm-level disease management.

### 4.2. Evaluation of the Vaccine Efficacy During TiLV Outbreak in RAS

Given that TiLV was endemic and persistent within the RAS on the farm in this case study, we evaluated the impact of the CNS4 vaccine [28]. Vaccination with CNS4 significantly reduced fish mortality: It achieved an RPS of 55.57% and lowered the daily mortality rates in the vaccinated groups compared with the unvaccinated groups. The protective efficacy of the CNS4 vaccine was accompanied by a concurrent reduction in TiLV concentrations in both the fish liver tissues and rearing water, which indicated that vaccination not only improved the host survival but also suppressed viral replication and shedding into the environment. This consistent pattern was observed across the five replicate ponds with vaccinated groups, which further emphasizes the reproducibility of the vaccine’s efficacy under commercial RAS conditions. Importantly, our study demonstrates an additional benefit of vaccination beyond individual protection, namely, its capacity to lower environmental viral loads and thereby reduce infection pressure within the culture system [48,49]. This environmental aspect of TiLV dynamics has not previously been explored and was likely influenced by vaccine-induced humoral and mucosal immune responses, including the generation of neutralizing antibodies, as shown in earlier TiLV vaccine trials [50,51]. Moreover, we did not observe any negative impact of vaccination on tilapia growth; fish in the vaccinated ponds showed similar size and weight compared with fish in the unvaccinated pond. The application of ddPCR to quantify the TiLV RNA in the water following vaccination in our study demonstrates that molecular monitoring can serve as an indirect indicator of vaccine performance in RAS conditions. Because water is continuously reused in RAS facilities, lowering environmental viral loads through vaccination could substantially reduce the risk of re-exposure and strengthen the overall biosecurity of such farms.

### 4.3. UV Efficiency in This RAS-Operated Tilapia Farms

In this study, the TiLV RNA concentrations in the RAS water frequently exceeded 3 log_10_ copies per 100 mL in three of the four experimental groups. The detection of TiLV in the circulating water prior to fish stocking in the farm suggests that the existing water treatment measures were insufficient to achieve complete virus removal. This remaining TiLV in the water can potentially contribute to the recurrence and severity of outbreaks, which has also been reported in other RAS facilities [46,52]. UV disinfection is widely applied in RAS operations to control microbial contamination, yet its efficiency depends on multiple factors, including the UV intensity and exposure time and the concentration of organic matter in the system [53]. For example, a UV dose of 100 mJ/cm^2^ has been shown to completely suppress *Piscine orthoreovirus* in salmonid farms [54], while doses of UV at 25 mJ/cm^2^ and 126 mJ/cm^2^ achieved 5.36 and 3.15 log_10_ reductions, respectively, of *Flavobacterium oreochromis* and *Aeromonas salmonicida* [55]. Likewise, in bivalve hatchery systems, UV exposure at 94 mJ/cm^2^ effectively inhibited the protozoan parasites *Perkinsus olseni* and *Bonamia ostreae* [56]. Although the farm in this study applied a UV exposure of approximately 50 mJ/cm^2^, the consistent detection of TiLV in the pre-stocking water suggests that this level of disinfection was operationally insufficient. Upon inspection, several factors were identified that likely contributed to the reduced disinfection performance: excess flow through the UV unit (shortened contact time), non-uniform hydraulics limiting the water–lamp contact, and damaged or non-functional lamps. Regular maintenance, flow-rate verification, lamp auditing/replacement, and the redesign of the UV chamber to optimize hydraulics and dose delivery are recommended to enhance disinfection efficiency and minimize environmental viral loads in RAS facilities. In parallel, controlled laboratory studies to determine TiLV-specific UV dose–response relationships are essential to generate basic data that can inform viral management strategies in RAS.

### 4.4. Comparison Between RAS and Non-RAS Operation

When the farm in our study temporarily shifted to a non-RAS operation, in which water was continuously discharged without recirculation, the TiLV concentrations in the water remained consistently lower than those recorded under the RAS condition throughout the study period. This finding supports the interpretation that viral accumulation in recirculating water is one of the key factors leading to elevated infection pressure in this farm. Combining other environmental factors such as the stocking density and water quality, these factors may act as stressors that synergize with viral accumulation to exacerbate outbreaks. Although continuous water exchange can effectively reduce environmental viral loads, it does not completely prevent infection, as has been reported for river-based cage systems, where fish are continuously exposed to pathogens from nearby infected farms [57,58]. Non-RAS facilities also incur substantial operational costs due to higher water and energy consumption and the need for wastewater treatment prior to discharge [59], which makes this type of setup unsustainable as a long-term biosecurity strategy. A more practical and sustainable solution thus involves integrating effective vaccination with optimized water treatment and disinfection protocols, along with routine environmental monitoring, to reduce viral recirculation and accumulation in rearing systems [60,61]. Such an integrated approach would enable farms to curb disease outbreaks, lower environmental viral loads, and strengthen overall biosecurity in RAS-based tilapia production.

### 4.5. Limitations and Future Perspectives

The findings of this case study have several limitations which limit the implications in other farms. Firstly, initial conditions of the fish, cleaning, disinfection, fallowing practices, and the viability of detected viral RNA were not fully established, and that these factors limit generalization towards other RAS facility. Specifically, the detection of TiLV in this study was based on molecular detection (qPCR), which quantifies viral genetic material but does not provide direct evidence of viral infectivity or viability. While the accumulation of TiLV RNA in water sample is strongly associated with the outbreak, this data is an indicator of contamination and shedding, not a definitive measure of infection pressure [62,63]. Future research should integrate viral isolation techniques or viability PCR to confirm the infectivity status of the detected environmental TiLV. Moreover, the system assessed here, while representative of a commercial operation, experienced conditions that could affect generalizability. Specifically, environmental control components such as the UV disinfection unit may not have operated at peak efficiency due to maintenance factors, lamp aging, or system conditions, which can influence pathogen inactivation and water quality outcomes [22,64]. Consequently, these findings primarily illustrate the risks and challenges associated with suboptimal RAS management during an active outbreak, rather than an inherent limitation of RAS technology itself. Caution is advised when comparing these results to facilities with different design specifications or more rigorous, documented maintenance protocols.

## 5. Conclusions

Field investigations at a commercial RAS showed that integrating vaccination with water-based molecular monitoring provided an effective strategy for managing TiLV outbreaks. Tracking TiLV RNA using the sensitive molecular tool ddPCR revealed that the viral concentrations in the water closely mirrored the infection dynamics and mortality patterns in the fish and thereby confirmed that environmental surveillance can provide valuable information on disease progression. Vaccination markedly improved fish survival, as demonstrated by an RPS of 55.6%, and reduced the TiLV loads in the fish and rearing water. These findings indicate that the vaccine not only protected individual fish but also helped lower the viral shedding and infection pressure within the RAS environment. When compared with non-RAS operations, the TiLV concentrations in the water were consistently higher under recirculating conditions, which suggests that water reuse can facilitate viral accumulation and elevate infection risk when maintenance and disinfection are inadequate. Proper management of RAS facilities is therefore essential both to maintain production and to prevent such systems from becoming reservoirs for persistent pathogens in intensive aquaculture settings.

## Figures and Tables

**Table 1:** Detection of TiLV RNA in pooled liver samples from Nile tilapia and water and sediment samples under outbreak (*n* = 16 per sample type) and no-outbreak conditions (*n* = 12 per sample type). The data are shown as mean ± standard error of the mean (SEM). The limits of detection (LOD) of the assay were 12 copies per µg of RNA for the fish tissue, 36 copies per 100 mL for the water samples, and 60 copies per 100 mg for the sediment samples.

Group	% Cumulative Mortality	TiLV RNA Concentration		
		Fish Copies/µg RNA	Water Copies/100 mL	Sediment Copies/100 mg
Outbreak (*n* = 16 each)	23.48% ± 3.60%	46,770.40 ± 28,511.28	7868.00 ± 3844.98	1395.22 ± 1354.60
No-outbreak (*n* = 12 each)	4.18% ± 0.54%	21.30 ± 3.20	194.50 ± 41.17	158.60 ± 24.33
	*p* = 0.0006 ^a^	*p* = 0.0438 ^b^	*p* = 0.0223 ^b^	*p* = 0.3265 ^b^

^a^ Two sample *t*-test; ^b^ Non-detects analysis using the generalized Wilcoxon test with Holm’s correction.

**Table 2:** Detection of TiLV RNA in the pooled liver samples and water and sediment samples from the unvaccinated and vaccinated groups during a TiLV outbreak (*n* = 8 per sample type per group). The data are presented as mean ± SEM. The LOD of the assay was 30 copies per µg of RNA for the liver samples, 36 copies per 100 mL for the water samples, and 60 copies per 100 mg for the sediment samples.

Group	% Cumulative Mortality	TiLV RNA Concentration		
		Fish Copies/µg RNA	Water Copies/100 mL	Sediment Copies/100 mg
Unvaccinated (*n* = 8 each)	37.65% ± 4.91%	811,899.90 ± 398,273.30	28,908.00 ± 11,476.50	13,695.00 ± 6995.08
Vaccinated (*n* = 8 each)	16.73% ± 1.03%	471.90 ± 411.64	278.00 ± 76.42	37,321.00 ± 14,535.76
	*p* = 0.0090 ^a^	*p* = 0.1172 ^b^	*p* = 0.0039 ^b^	*p* = 0.3265 ^b^

^a^ Two sample *t*-test; ^b^ Non-detects analysis using the generalized Wilcoxon test with Holm’s correction.

**Table 3:** Detection of TiLV RNA in the livers of individual fish samples (*n* = 80) and water (*n* = 8) and sediment (*n* = 8) samples during an outbreak among Nile tilapia under a non-RAS condition, compared with the RAS condition from the Experiment I. The data are presented as mean ± SEM. The LOD of the assay was 30 copies per µg of RNA for the liver samples, 36 copies per 100 mL for the water samples, and 60 copies per 100 mg for the sediment samples.

Group	% Cumulative Mortality	TiLV RNA Concentrations		
		Fish Copies/µg RNA	Water Copies/100 mL	Sediment Copies/100 mg
Outbreak non-RAS	17.38% ± 4.74%	64,136.60 ± 29,782.51	92.30 ± 50.90	188.80 ± 52.40
Outbreak RAS	23.48% ± 3.60%	46,770.40 ± 28,511.28	7868.00 ± 3844.98	1395.22 ± 1354.60
	*p* = 0.3442 ^a^	*p* = 0.9415 ^b^	*p* = 0.0064 ^b^	*p* = 0.8336 ^b^

^a^ Two sample *t*-test; ^b^ Non-detects analysis using the generalized Wilcoxon test with Holm’s correction.

## Data Availability

The original contributions presented in this study are included in the article/Supplementary Material. Further inquiries can be directed to the corresponding authors.

## Supplementary Materials

The following supporting information can be downloaded at: https://www.mdpi.com/article/10.3390/v18010096/s1, Table S1: TiLV concentrations in baseline (pre-stocking) and rearing pond water collected on days 1, 4, 7, and 14 after daily mortality exceeded 1% under RAS and non-RAS conditions.
