# Supplementary material for: Dynamics of Tilapia Lake Virus in Recirculating Aquaculture Systems and the Impact of Vaccination on Outbreak Control
Source: Viruses. 2026 Jan 9;18(1):96. doi: 10.3390/v18010096 (PMC12846342; doi:10.3390/v18010096)
Supplement: Supplementary file 1 [file viruses-18-00096-s001.zip › viruses-4007546-supplementary.pdf]

Supplementary Table S1. TiLV concentrations in baseline (pre-stocking) and rearing pond water collected on days 1, 4, 7, and 14 after daily mortality exceeded 1% under RAS and non-RAS conditions. The data are presented as mean ± SEM. The LOD of the assay was 1.56 $\log_{10}$ copies per 100 mL.

**Experimental conditions#**	**TiLV viral load in water** ($\log_{10}$ copies per 100 mL)			**Comparison with pre-stocking** (*p*-value) [a]
	Pre-stocking baseline	Sampling time point	Fish rearing pond	
**RAS: no-outbreak**	3.02	1	2.54 ± 1.68	0.0021
		4	1.92 ± 1.46	<0.0001
		7	2.18 ± 1.73	0.0015
**RAS: outbreak**	<LOD	1	4.19 ± 4.15	0.8093
		4	4.03 ± 3.88	0.6759
		7	3.41 ± 3.28	0.7094
		14	3.43 ± 3.18	0.5522
**RAS: outbreak unvaccinated**	3.13	1	4.44 ± 3.84	0.3253
		4	4.73 ± 4.70	0.8358
		7	4.44 ± 3.18	0.0754
		14	3.84 ± 1.48	0.0211
**RAS: outbreak vaccinated**	3.13	1	2.80	0.3540
		4	2.50 ± 2.06	0.1447
		7	2.56 ± 2.10	0.1648
		14	1.92 ± 1.26	0.0211
**No RAS outbreak**	<LOD	1	2.39 ± 2.25	NA
		4	<LOD	NA
		7	2.62	NA
		14	<LOD	NA

[a] Repeated measures ANOVA followed by Tukey’s multiple comparisons test.
